# Differential DNA-methylation of synaptic genes in CSF and blood in schizophrenia

**DOI:** 10.1038/s41537-026-00738-x

**Published:** 2026-02-20

**Authors:** Kirsten Jahn, Adrian Groh, Ole Riemer, Sunyu Sun, Nicole Moschny, Stefan Bleich, Helge Frieling, Franz Felix Konen, Thomas Skripuletz

**Affiliations:** 1 Laboratory of Molecular Neurosciences (LMN), Dept. of Psychiatry, Social Psychiatry and Psychotherapy, Hannover Medical School, Hannover, Germany; 2 Dept. of Neurology, Hannover Medical School, Hannover, Germany

**Keywords:** Epigenetics in the nervous system, Schizophrenia, Synaptic transmission, Biomarkers

## Abstract

The dual-hit model of schizophrenic psychoses suggests that epigenetic alterations may contribute to the disease pathogenesis. Given the significant synaptic loss in patients with schizophrenia (SZ) during puberty, we investigated DNA-methylation patterns of key synaptic target molecules: dopamine transporter (DAT), dopamine receptor D2 (DRD2), microtubule-associated protein tau (MAPT), and postsynaptic density protein 95 (PSD95). Analyses were performed in both blood and cerebrospinal fluid (CSF) samples from patients with SZ (n = 36) and healthy controls (Co) (n = 23). Due to the minimal amount of cell-free DNA available in CSF, different extraction methods were evaluated to achieve the best possible recovery. Ultimately, an adapted ethanol-glycogen precipitation protocol combined with a subsequent bead-based fusion and DNA clean-up was applied. However, despite comparable DNA concentrations obtained from Co and SZ CSF samples, only very few sequences could be obtained from CSF samples of Co, so that results concerning CSF measurements are limited to patients with SZ. In DAT, methylation was significantly higher in the blood of Co compared to both the blood and CSF of patients with SZ. In PSD95, mean methylation levels were higher in the CSF than in the blood of patients with SZ, whereas no difference was detected in the blood between SZ and Co. For MAPT and DRD2, no significant differences in mean methylation rates were observed between groups. Low sequencing success in CSF from Co, despite comparable concentrations to SZ, might point to a higher degree of fragmentation. In SZ, longer DNA fragments may be replenished more frequently. Higher central methylation of PSD95 in patients with SZ, a key regulator of glutamatergic neurotransmission, may reduce gene transcription and thus support the glutamate hypothesis of SZ, which assumes impaired glutamate receptor function. Lower DAT methylation in SZ compared to Co (with similar central and peripheral levels) could indicate a higher availability of the transporter at the synapse in SZ, resulting in a higher clearance of dopamine. This could be a compensatory mechanism concerning the hypothesis of dopaminergic hyperactivity in SZ.

## Introduction

Today, it is widely accepted that external stressors can increase the risk for the onset of psychiatric diseases like schizophrenia (SZ). In more detail, the “dual-hit model” assumes that there is a certain susceptibility in some individuals for the later development of SZ, which might be caused by a “first hit”, such as inborn subtle and symptomless changes in brain development. Later, environmental triggers, including trauma, substance abuse, or other stressors (the “second hit”), may aggravate the situation and lead to the onset of symptoms^1^. Environmental conditions can exert long-term effects on gene regulation by altering epigenetic mechanisms, particularly DNA methylation, without changing the underlying DNA sequence^2^. Even though there is a familial accumulation of SZ, it does not segregate in a Mendelian manner, which also supports the involvement of epigenetic mechanisms^3^. Functionally relevant DNA methylation occurs at cytosines positioned 5’ to guanosine (CpGs)^4^ and is one of the main regulatory epigenetic modifications^5^. In most cases, DNA-methylation impairs transcription factor binding in the CpG-rich promoter region of a gene^6^, thereby reducing its transcription.

It is known that approximately 30% of synapses are physiologically lost during puberty, whereas in patients with SZ this loss is significantly higher, reaching about 60%.^7^ Based on this, we investigated the methylation pattern of synapse-related genes in SZ and compared it to that of healthy controls. To assess potential differences between central and peripheral compartments, analyses were performed not only in blood but also in CSF. As brain tissue cannot be studied in living individuals, cell-free DNA (cf-DNA) from CSF represents the material closest to the brain. Importantly, there is no strict brain-CSF barrier, since exchange between the brain intercellular space and CSF occurs through several routes. Although CSF is mainly secreted by the choroid plexuses, there is also a direct exchange with the brain interstitial fluid, and more indirectly via the ependyma^8–10^. Furthermore, discontinuities in the ependyma of the ventricles do occur frequently in healthy individuals and patients with SZ in *post-mortem* investigations^11^. Thus, CSF provides a feasible access to brain-derived DNA in living individuals. In perspective, altered DNA-methylation patterns could even serve as a biomarker and could be implemented in the diagnostics of SZ.

In general, cfDNA is thought to be released during cell death (necrosis, apoptosis) or via exocytosis/extracellular vesicles^12^. cfDNA has been shown to have an average length of approximately 167 base pairs (bp), ranging from 10 bp to 1,200 bp, which corresponds to the length of DNA wrapped around Nucleosome Cores, whereas 10 bp is what is usually left over after deoxyribonuclease (DNase) digestion. The half-life of cf-DNA is about 16 minutes to 2.5 hours^13,14^. Elevated cfDNA concentrations are commonly observed in conditions associated with high cellular turnover, such as tumours ^14^.

Up to now, dopaminergic dysfunction is thought to be one of the major contributors to the pathogenesis of SZ. Increased dopamine levels are one of the most consistent findings in the disease^15^, and drugs that block D2 receptors (DRD2) are very effective in treating the positive symptoms^16^. Alterations in the expression levels of DRD2 and the Dopamine Transporter (DAT) have been shown in the midbrain of post-mortem brains of patients with SZ ^17^.

Another important neurotransmitter system in the pathogenesis of SZ is the glutamatergic system. The first hint of an involvement of this system was the observation that drugs that block N-methyl-D-aspartate receptors (NMDA), like Phencyclidine (PCP), can induce psychotic symptoms in humans, resembling those of schizophrenic patients with SZ^18^. Furthermore, it is known that the glutamatergic system is essential for the maturation of synapses^19,20^. A very important molecule at glutamatergic synapses is Postsynaptic Density 95 (PSD95/DLG4), which has been implicated in synaptogenesis, neurodevelopment, and cognition/learning skills. Alterations in the expression of this protein at the postsynaptic membrane lead to alterations in the composition of NMDA/ α-Amino-3-hydroxy-5-methyl-4-isoxazolepropionic acid (AMPA) receptors at glutamatergic synapses and can impact spine morphology. It can even lead to silent synapses during important developmental stages of the brain. It has been strongly associated with SZ and autism in the past^21^, and there have been hints of altered expression of PSD95 in SZ^22^. Therefore, we investigated PSD95 as a representative for molecules involved in receptor clustering.

Apart from the neurotransmitter receptors and transporters, certain “cofactor” genes are prerequisites for functional neurotransmission, like components of the cell skeleton. As a representative of specific neuronal cell-skeleton molecules, we investigated Tau (MAPT). Though it is mainly known for its role in dementia, altered (epi-) genetics of MAPT have also been linked with an increased risk of developing schizophrenic psychoses^23–27^. Furthermore, often the progress of SZ is accompanied by cognitive decline, and MAPT has been strongly associated with cognitive deficits in the past^28^. As cognitive impairments are a big part of schizophrenic symptom complexes besides positive and negative symptoms, we also included this target molecule in our analyses.

This study aimed to address two hypotheses: 1. According to the dual-hit hypothesis, DNA methylation patterns of synapse-related genes, including PSD95 and MAPT, could be altered in patients with schizophrenic psychoses in comparison to healthy controls, referring to differential synaptic neurotransmission in SZ.

2. A comparison of DNA-methylation patterns between central and peripheral compartments could provide novel insights into the potential of cfDNA-based methylation signatures as biomarkers for disease mechanisms and diagnostics.

## Material and Methods

### Recruitment of study participants

Participants were recruited from the psychiatric and neurologic wards of the Hannover Medical School. Lumbar puncture was performed as part of the standard diagnostic work-up, and for the study, an additional 2-max. 5 ml was collected. Participation required written informed consent, and the study was approved by the Ethics Committee of Hannover Medical School (no. 5221). Controls were people who presented due to temporal paraesthesia, which could not be objectified, and patients with normal pressure hydrocephalus (NPH).

### Withdrawal of CSF and blood

Two scaled 15 ml tubes from Greiner® were used to collect the CSF for the normal routine CSF diagnostic lab (6-10 ml CSF) and the research lab (2–max. 5 ml), respectively. The research tube contained a low amount of EDTA to protect DNA from DNase activity and thus prevent degradation.

A serum and EDTA sample were drawn for the routine diagnostics as well as for the research part.

### First processing step and storage of CSF and blood

CSF was centrifuged at 1400 g for 10 min. to separate cells and supernatant with free DNA. The supernatant was aliquoted á 1 ml in 1.5 ml screw cap cryotubes from Bioplastics® (free-standing, extra low binding, # B91141U, caps: # B91300). Cells and aliquots were stored at -80 °C until further processing. EDTA blood was aliquoted a‘ 600 µl in Matrix tubes and stored at -80 °C. Serum was kept at room temperature for 45 min and then centrifuged at 1400 g for 10 min. It was aliquoted a‘ 500 µl and stored at -80 °C.

### DNA extraction from blood

DNA from 200 µl EDTA blood was extracted with the automated Nucleomag DNA from blood-Kit (Macherey Nagel®) according to the manufacturer’s protocol using a Biomek Nxp (Beckman Coulter®).

### Bisulfite conversion of DNA from blood

500 ng DNA from blood was bisulfite-converted using the automated EZ-96 DNA Methylation-Lightning MagPrep Kit (Zymo Research, # D5046, D5047) according to the manufacturer’s protocol using a Biomek Nxp (Beckman Coulter®).

### Measurement of DNA concentration

To determine the initial concentration of DNA in the CSF, the Denovix dsDNA Ultra High Sensitivity Kit (# 31DSDNA-UL1, Biozym) was used to reliably measure the very low amounts of DNA in the CSF, enabling the detection of DNA concentrations as low as 0.5 pg/µl to 300 pg/µl. After extraction of DNA, the QuBit dsDNA High Sensitivity Kit (Invitrogen, # 32854, currently ThermoScientific, # Q33230) was used which is approved for the measurement of DNA concentrations ranging from 5 pg/µl to 120 ng/µl.

### DNA extraction from CSF

In the first step, we tested different strategies to figure out how to achieve the highest percentage of recovery of the DNA amount during extraction (Supplementary Table 1). This is a crucial point in the case of CSF as the DNA concentration is as low as about 0.04 ng/µl (Fig. 2) in non-infectious and non-tumorous conditions and the amount of material is limited for ethical reasons.

**Fig. 1:**
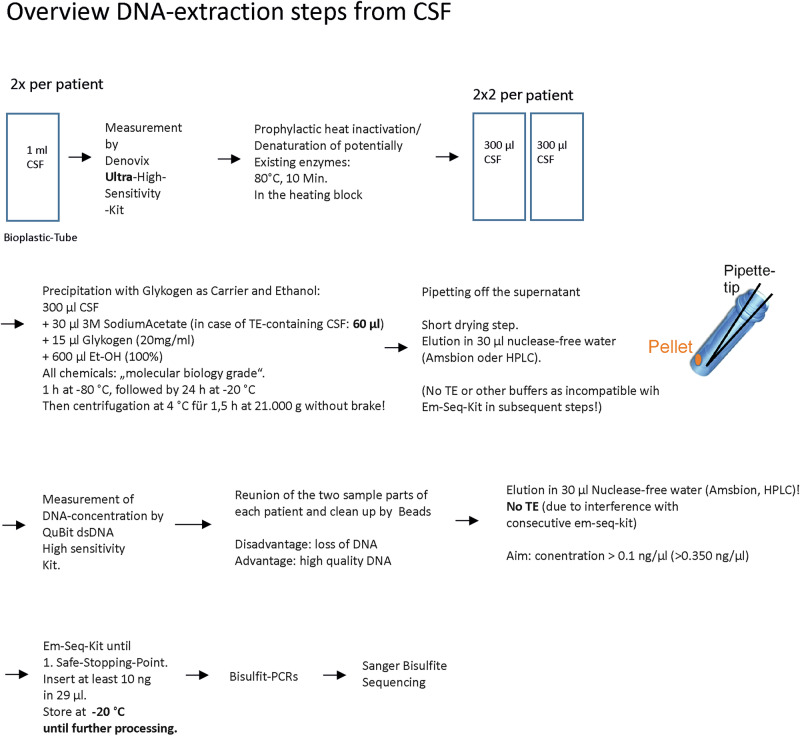
CSF DNA extraction steps. Overview of all steps of the DNA extraction from the CSF.

As also shown in Fig. 1, we finally used the following steps to extract DNA from CSF.

In general, sterile filter tips as well as sterile DNA-, DNAse-free ultra-low binding tubes were used. Personal equipment was gloves, face masks and coats to avoid contamination with external DNA. The working surface as well as pipettes were cleaned with “RNAseAway” before starting the extraction procedure.

Prior to extraction, the initial DNA concentration was measured using the Denovix Ultra-High Sensitivity Kit. Afterward, a heat inactivation step of potential DNases followed at 80 °C for 10 min to prevent DNA degradation. Subsequently, samples were split a‘ 300 µl. For further processing, 30 µl sodium acetate (Thermo Scientific, # R1181, 3 M), 15 µl Glycogene (molecular biology grade, thermos-scientific, #R0561, 20 mg/ml), and 600 µl 100% ethanol (molecular biology grade, VWR, #437433 T) were added to each sub-sample (all chemicals were molecular-grade). After mixing by slow and cautious pipetting up and down, samples were kept at -80 °C for 1 h, followed by 24 h at -20 °C. Then, samples were centrifuged at 4 °C for 1.5 h at 21,000 g without brake. Consecutively, the supernatant was carefully removed by pipetting under visual control of the pellet. For this purpose, the highest portion was removed using a 1 ml blue pipette tip, which was slowly moved from the surface of the supernatant to deeper regions of the tube. The supernatant closer to the pellet was removed with a 100 µl yellow pipette tip. The very last rests were removed with the help of a 10 µl grey pipette tip. Then, a short drying step followed. For elution, 30 µl nuclease-free water was added, and samples were kept for 10 min at room temperature (RT), followed by 10 min at 37 °C with a pipetting step in between. Again, the DNA concentration of samples was measured, now using the Denovix high-sensitivity kit. Consecutively, samples were processed until the first save stopping point of the em-seq kit (for gentle bisulfite conversion of very low DNA amounts) and kept at -20 °C until further processing.

### Bisulfite Conversion of DNA from CSF

The DNA from the CSF was bisulfite-converted by means of the EM-seq Kit (NEBNext Enzymatic Methyl-seq, Macherey Nagel®), following the manufacturer’s instructions. The Em-Seq Kit allows very gentle DNA conversion with almost no risk of losing DNA, and is especially suitable for the conversion of low DNA amounts. However, because its price per sample is almost 18 times that of the EZ-96 DNA Methylation-Lightning Mag Prep Kit from Zymo Research, we used the Zymo Kit for the blood samples, which is also better suited to higher DNA amounts.

### PCRs and sequencing

#### Methylation analysis

In order to amplify the sequences of interest, (semi-) nested polymerase chain reactions (PCRs) were performed by use of the HotStarTaq Master Mix Kit (Qiagen, 203445). According to the manufacturer’s protocol, the reaction mix was composed of the following: 1 µl bisulfite-converted DNA, either from the em-seq kit (in the case of CSF-derived DNA) or via the EZ-96 DNA Methylation-Lightning MagPrep Kit (in the case of blood-derived DNA), (or 1 µl of PCR1 product in case of preparing a consecutive second PCR), 5 µl HotStarTaq Master Mix Kit, 0.4 µl primer forward (20 pmol/µl), 0.4 µl primer reverse (20 pmol/µl) and 3.2 µl nuclease-free water. Primer sequences, the position of analysed targets, as well as the number of analysed CpGs and PCR temperatures are provided in Supplementary Table 2. As deducible from the base positions, some of the targets had rather long PCR products (for example, 731 Bp in the case of PSD95). Although this might reduce the probability for successful sequencing when dealing with short DNA fragments, like in our case, it is also important to include as many CpGs from a CpG island as possible because the probability that the methylation of single CpGs has an impact on expression is rather low. However, to check if there are long enough fragments for the analyses, we performed some long PCRs (734 bp) in the genomic DNA in some test samples before bisulfite conversion, and got visible PCR products in the gel. The Sequencing PCR and Sequencing according to Sanger were performed using the BigDye Terminator v3.1 Cycle Sequencing Kit (Applied Biosystems, 4337455). The reaction mix for the sequencing PCR was composed of about 30 ng (max. 6.9 µl) PCR-product, 0.5 µl Big Dye 3.1, 2.0 µl Big Dye Buffer, 0.6 µl primer forward or reverse (5 pmol/µl) and x µl nuclease free water to obtain a total volume of 10 µl (BigDye® Terminator v3.1 Sequencing Kit (Applied Biosystems, Foster City, CA, USA, 4337455)). Parameters for the (semi-) nested touchdown-PCR were chosen like following: 95 °C for 15 min (min), 97 °C for 2 min, followed by 15 cycles of 95 °C for 30 seconds (sec), X°C (temperature depending on target, see Table 3) for 45 s and 68 °C for 1 min, followed by 15 cycles of 95 °C for 30 s, X°C-15 °C for 90 s and 68 °C for 2 min. Samples were then kept at 68 °C for 4 min and afterwards at 12 °C until they were removed from the cycler. Settings for the sequencing PCR protocol were 96 °C for 60 sec., followed by 28 cycles of 96 °C for 10 s, 50 °C for 5 sec. and 60 °C for 4 min. Samples were then kept at 12 °C in the cycler until further processing. The products from the (semi-) nested touchdown-PCR were purified using Agencourt®AMPure®XP beads (Beckman Coulter GmbH, Krefeld, Germany, A63881) on a Biomek MC96 (Beckman Coulter GmbH, Krefeld, Germany). For the clean-up of sequencing PCR products, Agencourt®CleanSeq® beads (Beckman Coulter GmbH, Krefeld, Germany, A29154) were used on a Biomek MC96 (Beckman Coulter GmbH, Krefeld, Germany). Sanger bisulfite sequencing was performed on an Applied Biosystems ® 3500 xL DNA Analyser (Applied Biosystems, Foster City, CA, USA) in 15 µl HIDI (Formamide, Applied Biosystems, 4311320).

### Methylation Data Analysis

Before analyses, the raw sequencing traces were checked by Sequence Scanner Software 2, and only traces with a QV > 20 were considered for analyses. Representative sequencing electrograms are given in Supplementary Fig. 1.

In a first step, sequence trace files were processed by the ESME (Epigenetic Sequencing Methylation) software package in order to determine the DNA methylation rate. “ESME” automatically normalises signals, corrects for incomplete bisulfite conversion, and executes a quality control^29^. According to the common consensus, base positions are labelled negative in promoter regions and positive from the beginning of the first exon, while the first base of the first exon corresponds to position zero.

### Statistics

Statistical analysis of methylation rates was performed by usage of IBM SPSS Statistics 29 (IBM, New York, NY). In case data was available for all three triplicates or at least in two of the triplicates, the average of the values was calculated for the respective sample. To compare the mean DNA methylation between the different conditions (CSF vs. blood and SZ vs. Co) the linear mixed model was used. Data are given as mean ± S.E.M. Differences with p-values < 0.05 were considered significant. In general, different models of analysis of variance can be developed using the mixed linear model in SPSS. The mixed linear model can handle a large number of variables and covariates. The level of significance was set at *p* ≤ 0.05 throughout the analysis while correcting for multiple comparisons for the post-hoc t-tests (Bonferroni). Age and gender were used as covariates.

### Correlation analyses

Correlation analyses were done for the following relations: cell count and concentration of cell-free DNA, group membership and sequencing outcome as well as for medication (chlorpromazine equivalent (CPZ)) and methylation levels. For this purpose, a Pearson correlation analysis was performed.

### Calculation of CPZ equivalents

CPZ is the amount of Chlorpromazine that equals the effects of 1 mg of the respective substance. Therefore, corresponding CPZ equivalents were multiplied by the applied daily amount of the respective neuroleptic drug. In case of a combination of multiple drugs, the sum of the products was calculated for each patient.

### Ethics approval and consent to participate

The study protocol was reviewed and approved by the Hannover Medical School ethics committee (ethics approval no. 5221). All study participants gave their written informed consent to participate in the study.

## Results

### Demographics

Originally, CSF and blood samples were obtained from 50 patients with SZ and 49 healthy controls (Co). After excluding individuals with potential confounding factors such as chronic infections (e.g., treated HIV) or other conditions potentially interfering with DNA methylation, and matching of the groups for age, 59 participants (36 SZ and 23 Co) remained for the final investigation. The respective distribution of age and sex is presented in Table 1.

**Table 1:** Demographics of study participants.

Group	Sex (count)	Age (years)
SZ	M 21	32,4
	F 15	33,9
Co	M 10	29,2
	F 13	35,6

### DNA concentration and sequencing outcome

DNA was extracted from 1.2 ml of CSF and 200 µl of whole EDTA blood. As expected, there were only very low amounts of cell-free DNA in the CSF samples, about 0.04 ng/µl (+/- 0.02), for both Co and patients with SZ as measured by the Denovix dsDNA ultra-high sensitivity kit.

**Fig. 2:**
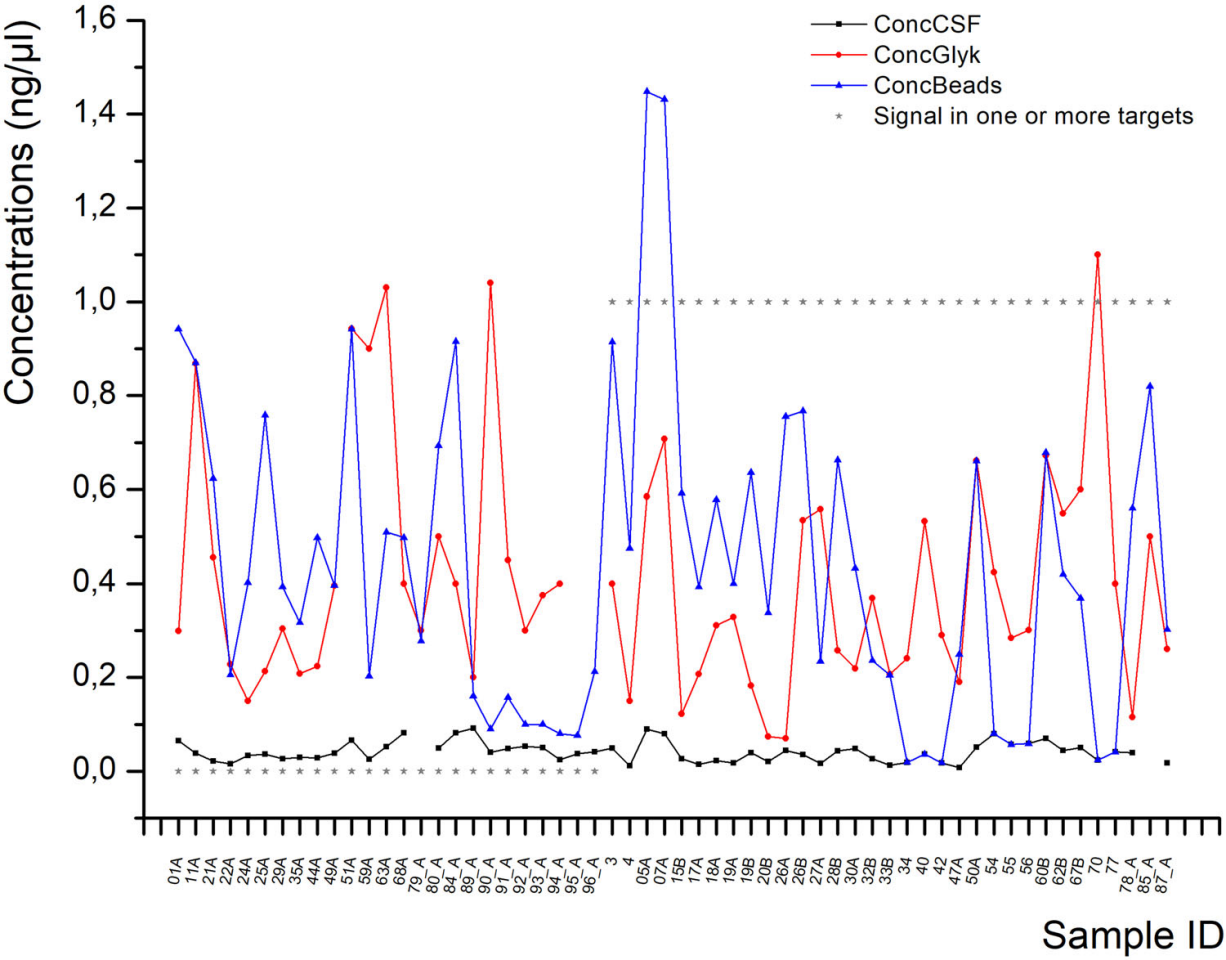
DNA concentrations at different stages of the DNA extraction from the CSF. DNA concentration in CSF before DNA extraction (black dots), after ethanol-glycogene extraction (red dots), and after bead-based purification and merging of the portions of the same sample (blue dots). Grey stars indicate whether PCR and sequencing were successful (1) or not (0).

Figure 2 shows the concentration of DNA in CSF before extraction (black line, in average 0.04 ng/µl +/- 0.02), after ethanol-glycogen-precipitation (red line, in average 0.41 ng/µl +/- 0.25), and after merging two portions of one sample during bead-based purification (blue colour, 0.44 ng/µl +/-0.34).

It can easily be seen that fluctuations in the final concentration had no impact on the outcome, if a PCR/Sequencing signal could be detected in individual samples or not.

### DNA concentration and sequencing outcome, calculated separately for groups

The independence of DNA concentration and sequencing outcome also becomes clear when looking at the average DNA amount subdivided by PCR/Sequencing signal yes/no for each group (Co and SZ) as the average DNA concentration was partly even lower in samples that generated a PCR signal as compared to those that did not (Table 2). Another noticeable finding is that the average DNA concentration after the junction of two portions and consecutive bead-based clean-up is lower in the case of Co (0.28 + /- 0.24 ng/µl) and increased in the case of SZ (0.53 + /- 0.35 ng/µl) (Table 2). Elution volumes were the same after the two steps.

**Table 2:** DNA concentrations (mean +/- SEM and n-numbers) in ng/µl in CSF at different time points of the extraction procedure for both groups of study participants and additionally separated by sequencing outcome.

	CSF			Glycogene			Beads		
	Average	PCR/Seq Signal		Average	PCR/Seq Signal		Average	PCR/Seq Signal	
		Yes	No		Yes	No		Yes	No
Co	0.04 + /-0.02	0.03 + /- 0.01, *n* = 9	0.05 + /- 0.02, *n* = 14	0.46 + /- 0.27	0.48 + /- 0.27, *n* = 9	0.44 + /- 0.27, *n* = 14	0.28 + /- 0.24	0.16 + /- 0.17, *n* = 9	0.36 + /- 0.26, *n* = 14
SZ	0.04 + /- 0.02	0.04 + /-0.02, *n* = 24	0.04 + /- 0.02, *n* = 10	0.4 + /- 0.24	0.34 + /- 0.2, *n* = 24	0.48 + /- 0.3, *n* = 10	0.53 + /- 0.35	0.56 + /- 0.36, *n* = 24	0.48 + /- 0.33, *n* = 10

### Correlation of cell count and DNA concentration

To exclude any artifacts in DNA concentration caused by differences in the lumbar puncture skills, the cell count before the centrifugation step has been correlated with the concentration of DNA. The Pearson correlation coefficient was 0.019 (*p* = 0.916), revealing no correlation between CSF cell number and DNA concentration.

### Correlation of group and sequencing outcome

**Fig. 3:**
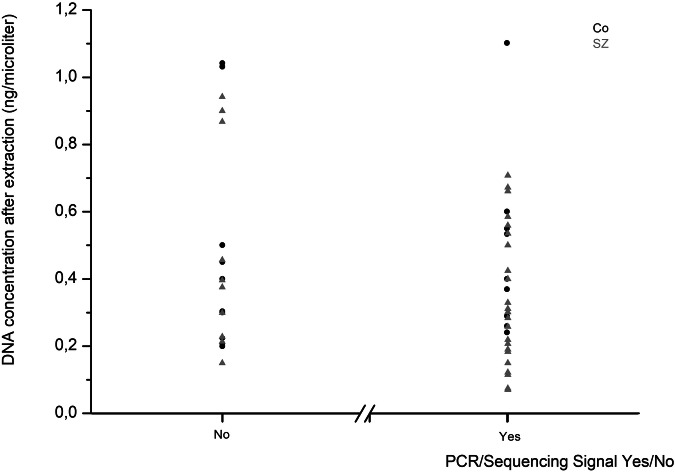
Sequencing success in relation to the DNA concentration. Overview of the number of samples for which PCR and subsequent sequencing were successful in at least one target vs. those that were not in relation to their DNA concentration after DNA extraction. Black dots: control samples, grey dots: SZ samples.

In the Co group, sequences were obtained in 40.9% of CSF samples, whereas in patients with SZ, the rate was 66.7% (Fig. 3). It has to be mentioned that these percentages refer to samples in which at least one target generated a signal. Therefore, success rates for individual targets were slightly lower. For DRD2, DAT, and PSD95, sequences were detected in only four or fewer Co CSF samples, precluding meaningful group comparisons for these targets. Signal generation did not correlate with DNA concentration in CSF prior to extraction (Pearson correlation coefficient: -0.164, *p* = 0.23), after precipitation (-0.166, *p* = 0.22), or after bead-based clean-up/junction (0.051, *p* = 0.7). An overview of which genes and compartments had adequate sequencing is provided in Supplementary Table 3.

### Sequencing results for MAPT, DRD2, DAT, and PSD 95

**Fig. 4:**
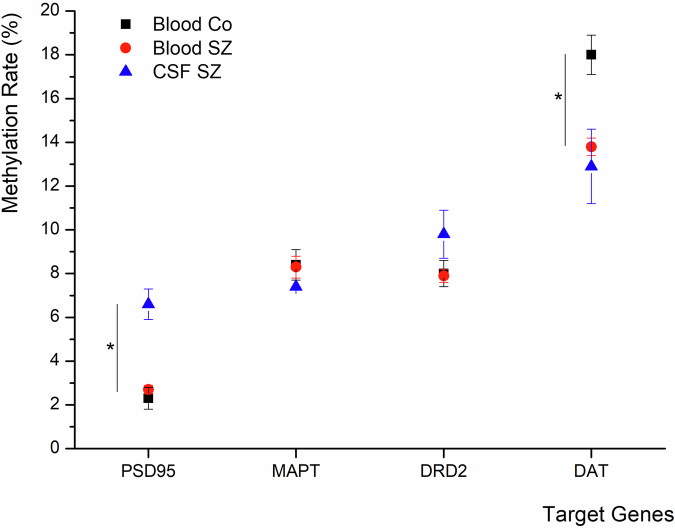
Mean DNA methylation levels in the different investigated targets and conditions. Comparison of mean methylation levels (%, + /- SEM) between different body fluids in controls and SZ in the different target genes, revealing a significant difference in methylation rates between different body fluids in the case of PSD95 and a significant difference in methylation rates between the different groups of study participants (SZ vs. Co) in the case of DAT.

Fig. 4 gives an overview of the methylation levels (in %), which will be described in the following for every target.

### MAPT

For MAPT, no significant group differences were observed in blood between Co (8.4 + /- 0.7%) and patients with SZ (8.3 + /- 0.5%). In CSF, methylation levels were slightly higher in Co (9.3 + /- 2.1%) compared to the SZ group (7.4 + /- 0.6%), but this difference did not reach statistical significance (*p* > 0.05).

Comparison between the two body fluids revealed in Co a mean methylation rate of 8.8 + /- 1.3% in blood and 9.2 + /- 1.2% in CSF, with no significant difference (*p* > 0.05). In the SZ group, mean methylation was 8.2 + /- 0.6% in blood and 7.4 + /- 0.5% in CSF, which likewise did not reach statistical significance.

### DRD2

As mentioned earlier, in DRD2, there were not enough sequencing data in Co CSF samples, disabling reliable analysis.

In blood, methylation rates did not differ significantly between Co (8.0 + /- 0.6%) and patients with SZ (7.9 + /- 0.3%); p > 0.05. For CSF, the mean methylation rate in SZ was 9.8% +/- 1.1%. As mentioned, there were no reliable values for Co in CSF.

Within the SZ group, comparison of blood (8.0 + /- 0.3%) and CSF (7.7 + /- 1.3%) also revealed no significant difference. In the control group, mean methylation in blood was 8.0% +/- 0.5% in Co, but there were no Co CSF for a proper comparison.

### DAT

In blood, comparison of mean methylation rates between Co (18.0 + /- 0.9%) and SZ (13.8 + /- 0.4%) revealed a highly significant group difference (*p* < 0.01). Due to the low number of Co CSF samples with sequences, no valid group comparison could be performed. However, in patients with SZ, the mean methylation rate in CSF was 12.9 ± 1.7%.

Within the SZ group, comparison between blood (13.8 ± 0.5%) and CSF (11.8 ± 1.3%) showed no significant difference. In Co, reliable CSF data were lacking, but mean methylation in blood was 17.4 ± 1.2%.

### PSD95

In blood, mean methylation rates did not differ significantly between Co (2.3 + /- 0.5%) and patients with SZ (2.7 + /- 0.2%). Due to the low number of Co CSF samples with sequences, no valid group comparison could be performed. Patients with SZ showed a mean methylation rate of 6.6 + /- 0.7% in the CSF.

Within the SZ group, there was a significant difference between mean methylation rates between blood (2.8 + /- 0.4%) and CSF (6.7 + /- 0.5%), p < 0.001. In Co, insufficient CSF data were available for analysis, while the mean methylation in blood was 2.6 ± 0.5%.

### Correlation of medication and methylation levels

As mentioned in the methods part, age and sex were used as covariates in the mixed linear model. Since the medication, i.e., especially neuroleptic drugs^30^, could also influence the methylation level, Chlorpromazine equivalents were calculated in order to standardise the different therapy strategies used in the patients of our study cohort and for better comparability of their neuroleptic potency. Supplementary Table 4 shows the used Chlorpromazine equivalent factors according to Rijcken et al., and Schmauß et al., 2019 ^31,32^.

However, it was not possible to use it as a covariate in the mixed linear model as it was constant in Co(0). Therefore, methylation rates were separately correlated with the Chlorpromazine equivalents for patients with SZ in every target. Correlation analyses revealed no significant correlations except with the level of DAT methylation in the blood. (Table 3).

**Table 3:** Correlation of Methylation Rates and medication (Chlorpromazine equivalents).

Target		Correlation Coefficient	p-value	n-number of data points for the calculation	
				CPZ equivalent	Methylation Rate (CpGs, Patients)
DAT	Blood	-0.17	<0.001 **	798	849
	CSF	0.11	0.074	280	302
DRD2	Blood	-0.025	0.181	3021	3112
	CSF	0.08	0.08	636	612
MAPT	Blood	0.009	0.8	714	741
	CSF	-0.031	0.303	1122	1168
PSD95	Blood	-0.024	0.46	1428	926
	CSF	-0.006	0.886	884	989

## Discussion

The present study provides first insights into DNA methylation patterns of synapse-related genes in cell-free DNA derived from CSF and blood of patients with SZ compared to healthy controls (Co).

As already mentioned in the introduction, the main study hypotheses were that 1. DNA methylation patterns of synapse-related genes, including PSD95 and MAPT, could be altered in patients with schizophrenic psychoses in comparison to healthy Co, referring to differential synaptic neurotransmission in SZ and the dual-hit hypothesis of SZ. 2. A comparison of DNA-methylation patterns between central and peripheral compartments could provide novel insights into the potential of cfDNA-based methylation signatures as biomarkers for disease mechanisms and diagnostics. Although it could be argued that a lumbar puncture is an invasive procedure, it must be kept in mind that it is part of the routine diagnostics in SZ.

Given the generally low concentrations of cfDNA in CSF, we first evaluated extraction strategies and sequencing efficiency before analysing target-specific methylation rates. While sequencing success was limited in Co CSF samples, likely due to stronger DNA fragmentation, several consistent patterns emerged in the patient group.

### DNA concentration and sequencing outcome

Baseline DNA concentration in the CSF of our study participants, measured with the Denovix Ultra-high sensitivity kit, was approximately 0.04 ng/µl with no major group differences. In other words about 40 ng per 1 ml CSF. This is in line with the findings of Afflerbach et al., who reported about 108 ng per ml CSF (ranging from 10 to 150 ng in 1 ml CSF), but in brain tumour patients^33^ using the High Sensitivity DNA assay on an Invitrogen Qubit 3.0 device. However, some other groups used different methods for the determination of the DNA concentration, such as PrimerPCR^TM^ Mutation assays, and obtained considerably lower values: for example, Ye et al. found 502 copies/ml in epilepsy patients, corresponding to ~1.5 ng/ml (0.0015 ng/µl), i.e., ~25-fold less than in our cohort^34^. Despite methodological variability, all studies consistently show that cfDNA in CSF is present only at low concentrations, underlining the need for efficient extraction methods with high recovery rates.

In preliminary experiments, we tested different extraction methods and determined the respective recovery rates (Supplementary Table 1). We obtained the highest recovery rate by ethanol/glycogen precipitation followed by Bead-based clean-up. Ethanol outperformed isopropanol, and although the additional bead step entails some DNA loss, it was necessary to remove glycogen that could interfere with the downstream EM-seq procedure. Beads alone would have been feasible but required disproportionately high volumes, resulting in excessive costs. To compensate for losses, we processed four 300 µl CSF aliquots per subject and pooled them after precipitation. Interestingly, sequencing success was not strictly dependent on DNA concentration (Fig. 3), likely reflecting the unknown degree of fragmentation.

cf- DNA has been shown in different body fluids to have a length of approximately 167 Bp or multiples thereof, referring to the length of DNA wrapped around a single nucleosome. Nucleosome positioning can vary between different tissues and could therefore help to determine the cell-type of origin prospectively^35^. Besides the mononucleosomal (mncfDNA, 167 Bp), dinucleosomal (dncfDNA, 320 Bp), and trinucleosomal (tncfDNA, 480 Bp), also longer cf-DNA fragments of 3 Kbp in length have been described lately^36^. Tumour DNA can be shorter (10–20 Bp)^35^. All in all, the length of cf-DNA can therefore range from 10 to 3000 Bp and has a half-time of 16 minutes to 2.5 h^13,14,36,37^. Although in SZ, DNA amounts in CSF do not reach those in tumorigenic or inflammatory diseases, there are hints that there is a higher cell turnover in SZ compared to Co^38^. Accordingly, Lubotzky et al. found elevated brain-derived cell-free DNA in the plasma of SZ in comparison to Co^39^, supporting the hypothesis that longer DNA fragments may be replenished more frequently in this condition.

This is further consistent with our observation that bead-based clean-up and fusion (bead to sample ratio 1.5, excluding fragments <100 bp^40^) reduced DNA yield primarily in Co, suggesting higher fragmentation in Co CSF. As PCR products in our study ranged from 208–824 bp (Supplementary Table 2), this could explain the lower sequencing success in Co. However, even in pathological conditions, there can be fluctuations in which fractions of the genome are available in an individual sample at the specific time point when the lumbar puncture took place.

**Fig. 5:**
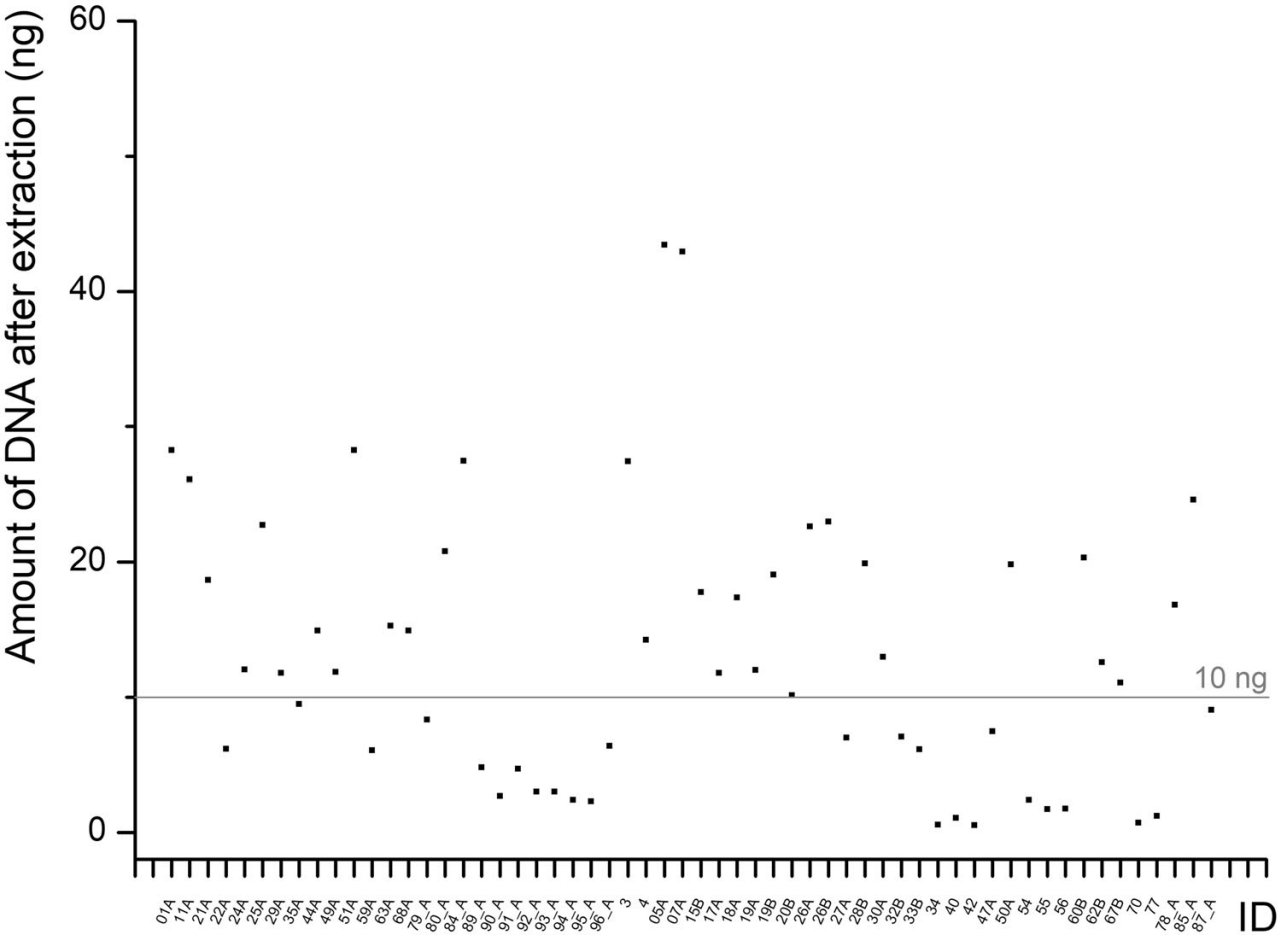
DNA amounts after DNA extraction from CSF. Available DNA amounts after extraction from ~1 ml CSF in the different individuals of our study cohort.

Nanopore sequencing, which allows whole-genome and methylome profiling even from shorter reads, was not feasible in our cohort due to insufficient DNA amounts. In the range of DNA fragment lengths that can be expected in our cohort, the respective kit (SQK-LSK110, Oxford Nanopore) recommends using at least 10 ng of DNA. In our cohort, for a high portion of samples, we had less (Fig. 5). Afflerbach et al^33^. successfully applied this technique in tumour patients, with DNA yields ranging from 1.5 to 3835 ng/ml CSF (mean ~108 ng/ml). In their cohort, input DNA ranged from 1.88 ng (failed) to 97.8 ng (mean 19.4 ng) per run. Importantly, even under their tumour conditions with higher cfDNA concentrations and larger CSF volumes, dropout rates remained substantial (62% success rate from 178 patients). This highlights the general challenge of sequencing cfDNA from CSF, particularly in non-tumour conditions such as SZ.

Furthermore, in our case, it did not make sense to amplify the whole DNA by whole genome amplification, as we were mainly interested in the methylation pattern, which this technique cannot transfer.

In order to obtain only cell-free DNA, we centrifuged the CSF and aliquoted the supernatant before freezing its aliquots. However, it could be argued that high cell counts, which could also depend on the punctuation abilities and skills, could lead to higher amounts of DNA in the CSF due to bursting cells during the acquisition procedure. To account for this possibility, we correlated the CSF cell count with the DNA concentration, but could not detect any correlation between CSF cell count and DNA concentration.

### Correlation of group and sequencing outcome

It was very conspicuous that only very few sequences could be obtained from Co CSF samples. For most targets, the number of Co samples with valid sequences was too low to allow proper analyses using the mixed linear model. There was almost no difference in the DNA content of the CSF samples between Co and SZ (Table 2). Therefore, the DNA content could not explain the drop-outs. As already discussed above, it is very probable that the DNA in the Co CSF had a higher degree of fragmentation. Accordingly, sequencing success could also not be correlated with the DNA concentration (Fig. 3).

### Sequencing results for MAPT, DRD2, DAT, and PSD 95

Regarding methylation in PSD95 and DRD2, rates were higher in CSF compared to blood within the SZ group, with the difference reaching significance for PSD95. In blood, methylation levels were almost similar between SZ and Co.

In contrast, methylation levels were almost similar in the blood and CSF of SZ in DAT. However, the blood methylation level of Co was significantly higher than in the blood of SZ.

In MAPT, there were no noticeable differences between groups.

In general, a lower DNA-methylation is linked to a higher expression level (negative correlation)^41^, and the following discussion of our results is based on this interrelation for our targets located in the promoter region and partly in exon 1. However, there can also be some exceptions: e.g., under pathological conditions such as cancer, positive correlations are to be found more frequently^42^. Sometimes, the effect also depends on the location of the methylation in the gene body (if positioned in the introns, it can have a positive correlation). Partly, it is also dependent on the sequence - some single-nucleotide polymorphisms (SNPs) can be associated with alterations in the methylation patterns ^43^.

Regarding our findings in the dopaminergic system, it is very interesting that there were almost no differences in methylation of the DRD2-receptor, but in the DAT. DAT was significantly lower methylated in the blood and CSF of SZ as compared to the blood of Co (difference was about 4%). As lower methylation is generally mostly associated with higher expression^44^, it might be assumed that the expression of DAT is at least facilitated in patients with SZ, which is reflected in lower central (CSF) and peripheral (blood) methylation. Regarding the general assumption of dopaminergic hyperactivity in SZ, a facilitated expression of DAT could be interpreted as compensatory since higher levels of DAT would lead to a faster clearance of dopamine at the synaptic cleft. As levels were comparable between CSF and blood, it could be hypothesised that peripheral DAT methylation status could be used as a kind of biomarker for central alterations. Nevertheless, there was a significant negative correlation of the DAT methylation level in blood with the CPZ equivalent, with higher CPZ equivalents leading to lower methylation levels (Table 3). In contrast, there was no significant correlation in CSF. However, the Pearson correlation coefficient of – 0.17 only indicates a very weak ( < 0.2) correlation. Anyways, it is also interesting that this potential effect of neuroleptic drugs mainly affects DAT and not DRD2.

When looking at PSD95, methylation of this target molecule was 4% higher in the CSF of SZ as compared to the CSF and blood of Co. PSD95 is known to have a great impact on the composition and function of glutamatergic synapses, as it is involved in synaptic maturation via interaction, stabilisation, and trafficking of NMDA- and AMPA-type glutamate receptors to the postsynaptic membrane. It has often been associated with SZ^21^. A higher central methylation compared to the peripheral status is per se no hint for something diagnosis-specific as we do not have the respective CSF values of Co. However, it is interesting that methylation is higher in CSF than in blood, since one would expect the expression to be higher centrally. This could point to a reduced availability in the brain which would be in line with the known impaired function of glutamatergic neurotransmission in the SZ. Inhibition of NMDA receptors by certain drugs like PCP can induce SZ-like psychotic episodes^45^. A lack of PSD95 could lead to an altered composition of glutamatergic synapses with less NMDA, in relation to AMPA-type glutamatergic receptors. However, it has to be kept in mind that there was no data for Co CSF, which limits the interpretation.

For MAPT, there were also data for CSF of Co available (not shown in Fig. 4 for better comprehensibility). However, there was no significant difference in either group comparison. Whereas there was almost no difference between the blood of Co and SZ at all, methylation was 2% higher in Co CSF as compared to SZ CSF. The relation of blood methylation levels to CSF methylation levels was therefore about 0.96 (8.8_blood_/9.2_CSF_) in Co. In SZ it was 1.1 (8.2_blood_/7.4_CSF_). Although the group comparisons did not reach the level of significance, this inversion of the ratio is quite interesting with regard to the fact that MAPT has been associated with an increased risk of developing schizophrenic psychoses^28^. In our findings, the ratio of blood methylation levels to CSF methylation levels shows the opposite direction in Co as compared to SZ, with a lower central methylation in SZ in trend, which could point to a higher expression of MAPT in SZ. It is hard to tell if this could be compensatory and/or if this leads to a disadvantageous accumulation, like observable in dementia. However, besides expression levels or mutations in MAPT^27,46^, also functional aspects like altered phosphorylation^28^ of MAPT seem to play an important role in the pathogenesis of SZ, which cannot be evaluated in the current study.

It is important to mention that all interpretations of the data have to be considered as hypothesis-generating since no reliable sequencing results could be produced in the CSF of Co (apart from the measurements in MAPT), probably resulting from higher fragmentation of the DNA as discussed in the previous section.

### Correlation of medication and methylation levels

Correlation analyses of methylation rates and chlorpromazine equivalents revealed only a very small ( < 0.2) significant correlation coefficient in the case of DAT in the blood samples of patients with SZ. For the other targets, no significant correlations could be detected. Although it could be argued that medication could have influenced the group comparison, it has to be kept in mind that the correlation coefficient was rather low. However, for the sake of completeness, Supplementary Fig. 2 shows a scatter plot for the data, revealing that there were fewer data points for high CPZ equivalents, which could also explain that higher methylation levels, which were less frequent at all CPZ equivalent dose ranges, no longer occurred.

### Limitations

The main limitation of the current study was the low number of valid CSF samples in Co, which is most likely inherent to the condition itself, as only very small amounts of longer cfDNA fragments can be expected in healthy individuals. Fragmentation analysis would have been helpful for interpretation, but it was not feasible due to the low DNA concentrations. Even other studies on cfDNA in CSF from tumour or inflammatory conditions could only determine fragment size distribution when DNA levels were sufficiently high ^33^.

As the analysis of nucleosome positioning for the identification of the cell origin of DNA is not yet fully developed, it was not possible to determine the cells from which our CSF DNA originates exactly. However, as we used centrifugation to separate the cf-DNA from the immune cells in the CSF, it is highly probable that the DNA originates from brain cells.

Another limitation is the use of different extraction strategies for blood and CSF. However, this was unavoidable given the distinct sample properties, and both approaches are known to be conservative and compatible with DNA methylation analyses. The high concordance of methylation rates between blood and CSF in unaffected targets further supports the comparability of both methods.

It could be argued that differences of about 5% in methylation are rather low and biological relevance could be questioned. However, the definition of DMRs (differentially methylated regions) in Whole Methylome Studies varies in dependence on the context, cell types and other conditions. In some studies, 2% is already considered a relevant difference. Others use 10 to 20%. To obtain any information about the impact of DNA-methylation differences, RNA expression would have been the most indicative parameter as it is the first expression product. Due to posttranscriptional activities, RNA expression and protein expression are often incoherent. However, there is almost no RNA in the CSF due to fast degradation. Furthermore, proteins of the CSF mostly stem from blood, as proteins can pass the blood-cerebrospinal fluid barrier. Therefore, their analyses would not have been correlatable with DNA-methylation.

Nanopore sequencing, which could have provided less bias (no DNA conversion- or PCR- bias) and broader genomic coverage, was not feasible under the current conditions. The last point could perspectively eventually help to clarify the exact cell type the DNA was arising from. Nevertheless, our protocol allowed the analysis of key target genes under very challenging conditions, i.e., in the CSF of patients with SZ, where cfDNA levels are much lower than in tumour or inflammatory diseases.

Finally, although we attempted to account for the potential effects of neuroleptic medication by calculating CPZ equivalents, this approach can only approximate the heterogeneity of therapeutic regimens. For future studies, it would therefore be desirable to investigate first-episode and therefore drug-naïve patients. For this purpose, multi-centre studies could be a promising approach.

## Summary and Conclusions

To our knowledge, this is the first study to determine methylation rates in cell-free DNA from CSF in patients with SZ in comparison to blood.

Our findings suggest higher central methylation of PSD95, a key regulator of glutamatergic neurotransmission, consistent with the glutamate hypothesis of SZ and potentially reflecting reduced glutamate receptor function. In contrast, lower methylation of DAT in SZ compared to Co points to increased transporter availability and enhanced dopamine clearance, which, within the framework of the dopaminergic hyperactivity hypothesis, may represent a compensatory mechanism.

These results highlight that cfDNA from CSF can provide insights into central epigenetic alterations in SZ. In perspective, such methylation patterns may contribute to a better understanding of disease mechanisms and could eventually hold potential as biomarkers for diagnosis or treatment monitoring.

However, there is still a need to develop improved low-input sequencing and to obtain larger CSF control cohorts in order to have enough material to reliably analyse the DNA methylation patterns also in the controls for a better central comparison.

## Supplementary information

Supplemental Material

## Data Availability

The data sets generated and analysed during the study are available from the corresponding author on reasonable request.
