# Supplementary material for: Differential DNA-methylation of synaptic genes in CSF and blood in schizophrenia
Source: Schizophrenia (Heidelb). 2026 Feb 20;12(1):30. doi: 10.1038/s41537-026-00738-x (PMC12993050; doi:10.1038/s41537-026-00738-x)
Supplement: Supplementary file 1 — Supplemental Material [file 41537_2026_738_MOESM1_ESM.pdf]

Supplemental Tables:

Category of DNA extraction technique	DNA-concentration (ng/µl) in CSF	Start Volume (µl) of CSF	First step	DNA-Recovery Rate (%)	Next Step	DNA-Recovery Rate (%) (from step before)
Speed Vac Concentration and column-based cleanup	0.04	1ml	SpeedVac (to 50µl)	72.5	QiaAMP DNA Mini Kit (for up to 50 Kb)	6
Speed Vac Concentration and column-based cleanup	0.04	1 ml	SpeedVac (to 200µl)	100	QiaAMP DNA Mini Kit (with AE-B and CarrierRNA) El-.Vol: 50 µl	25
Speed Vac Concentration and column-based cleanup	0.04	1ml	SpeedVac (to 20µl)	80	Nucleospin Plasma Kit (for 50 - 1000 Bp) El.-Vol.: 20 µl	0
Speed Vac Concentration and precipitation	0.04	1ml	A: SpeedVac (to 50µl)<br>B: -	A: 74<br>B: -	A: Gentra Pure Gene Kit<br>B: Gentra Pure Gene Kit	A: 72<br>B: 41
Bead-based DNA extraction with: AMPure	0.04	2 ml, partitioned at 300 or 600 µl	AMPure (El.- Vol: 30 µl)	65,5	SpeedVac (to 10 µl)	21
Bead-based DNA extraction with: CleanNGS	0.04	2 ml, partitioned at 300 or 600 µl	CleanNGS (El.-Vol: 30 µl)	74	SpeedVac (to 10 µl)	21
EtOH- Glycogene DNA Precipitation and Bead-based cleanup.	0.04	2 ml, partitioned at 300 or 600 µl	EtOH-Glycog. (El.-Vol: 30 µl)	100	Cleanup with Beads	80
Phenol- Chloroform DNA extraction	Not recommended for low DNA-concentration (common consent)					

Suppl. Table 1: Recovery rates of different DNA extraction techniques tested ahead of isolating the DNA from the cohort´s CSF samples

Gene	Gene Symbol/ Chromosome	Primers	CpGs were mapped between base positions:	Number of CpGs analysed in this region:	PCR Temperature X (°C)
PSD 95	DLG4/ 17	Fw: TGGGAATTTGATTTGTTTAGA<br>**Rev: TAACTACAATAACTTAAACCCCT**	- 607 to + 124	49 (to) 68	62 (TD)
NCAM (Neural Cell Adhesion Molecule)	NCAM1/ 11	Fw_a: AAGGAAGGTTGGGTAGTAGGA,<br>Fw_i: GGGTAGAAGTGTGAAAAG,<br>**Rev_i: ACAAACCTAAAAACAAACAAT**,<br>Rev_a: TCCCAAAAAAAACAAAATCCAA	-95 to +185	22	46
Dopamine Transporter	DAT/ 5	Fw: TTGTAGGTTGGAATGGTTG<br>Rev: CCTAAAAAAACCATTTCCC<br>**Seq: AAAAAATAAAACCCCC**	-1037 to -829	15	56 (TD)
Dopamine Receptor 2	DRD2/ 11	Fw_o: GTAATTTTTGGTTTTTGAGTT, Fw_i:GAGGAGGTATAGTTTTTTTGGT,<br>**Rev_i: CTACTTAAAACTTCCAACTCCC**, Rev_o:CACAAACTTCTAATCCTAACCT,	+132 to +461	53	62 (TD)
Tau (Microtubule Associated Protein Tau)	MAPT/ 17	Fw_o: TAATAAAAAAGGTGGGAAAAAA,<br>Fw_i: AAAGGAAGTAGTTTGGGG,<br>**Rev: AACCTCCCCAAAAAAAAAAA**	-337 to +27	49	70 (TD)

Suppl. Table 2: List of bisulfite primers, position of primers in relation to the first base of exon 1, number of analysed CpGs, and PCR temperatures. Primers used for sequencing PCR are highlighted in bold letters. TD: Touch Down PCR.

Gene	Blood SZ	Blood Co	CSF SZ	CSF Co
**PSD95**	36	23	19	1
**MAPT**	36	23	19	7
**DRD2**	36	23	20	2
**DAT**	36	23	23	4

Suppl. Table 3: Overview of the number of samples with adequate sequencing per group and gene. In the blood, numbers correspond to the number of participants per group because no dropouts occurred.

Neuroleptic drug	CPZ	Typical (T) /Atypical (A)
Acetophenazine	7,5	T
Amisulpiride	0,2	A
Aripiprazole	3,4	A
Butaperazine	12,5	T
Chlorpromazine	1	T
Chlorprothixene	1,5	T
Cariprazine	40	A
Clopenthixol	1,5	T
Clozapine	2	A
Flupentixol	74	T
Fluphenazine	65	T
Haloperidol	48	T
Levomepromazine	0,5	T
Loxapine	10	T
Mesoridazine	1	T
Molindone	7,5	T
Moperone	17	T
Olanzapine	40	A
Perazine	1	T
Periciazin	7,5	T
Perphenazine	10	T
Pimozide	65	T
Pipamperone	0,8	T
Prochlorperazine	5,5	T
Promazine	0,5	T
Quetiapine	0,5	A
Risperidone	89	A
Sertindole	40	A
Sulpiride	1	T
Thiopropazate	15	T
Thioproperazine	25	T
Thioridazine	1	T
Thiothixene	35	T
Tiapride	1	T
Trifluoperazine	22,5	T
Triflupromazine	3	T
Ziprasidone	1	A

Suppl. Table 4: Chlorpromazine equivalents used for the calculation of the neuroleptic potency of the different neuroleptic therapies that the SZ patients received.

Supplemental Figures:

MAPT

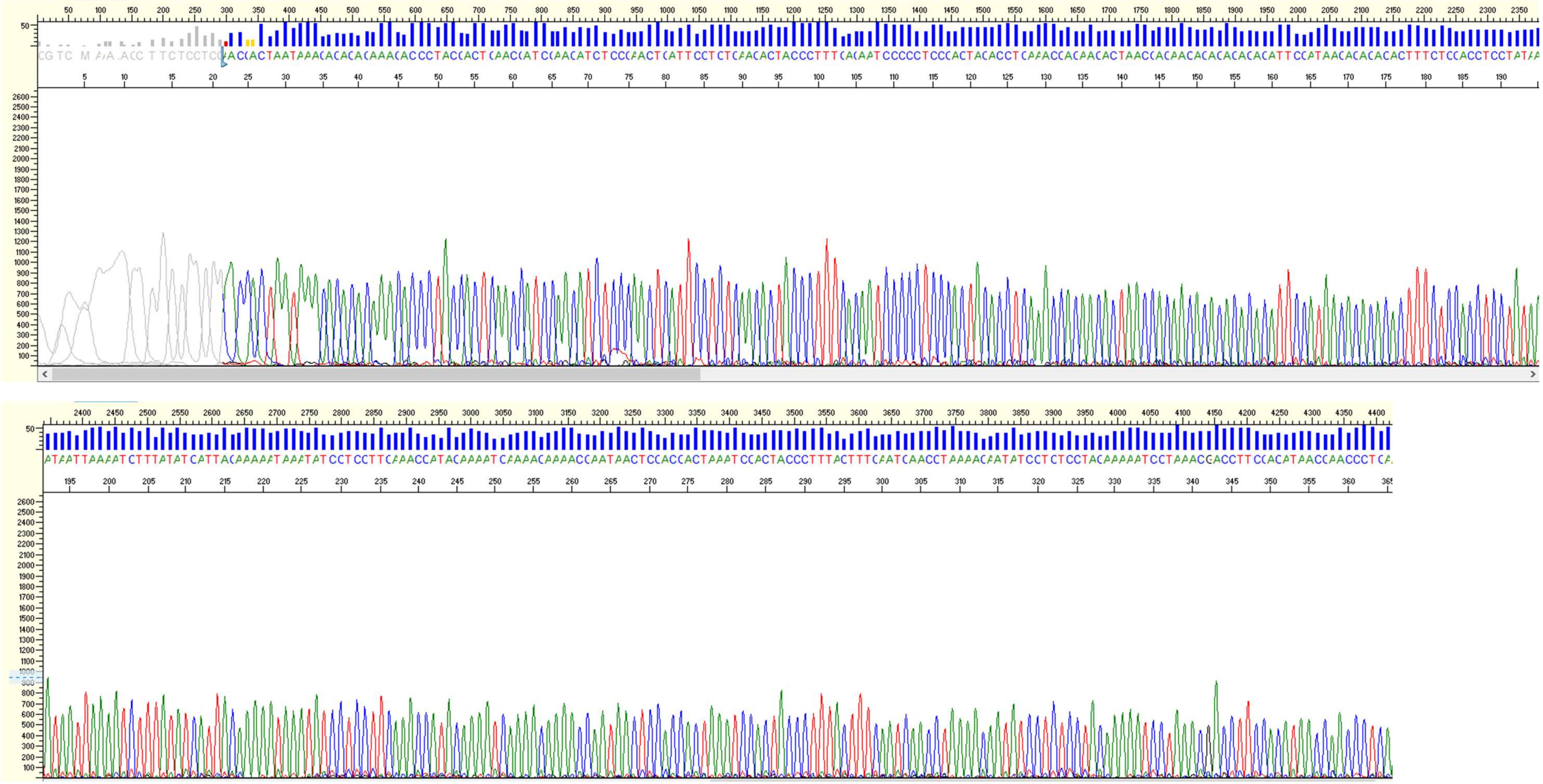

DRD2

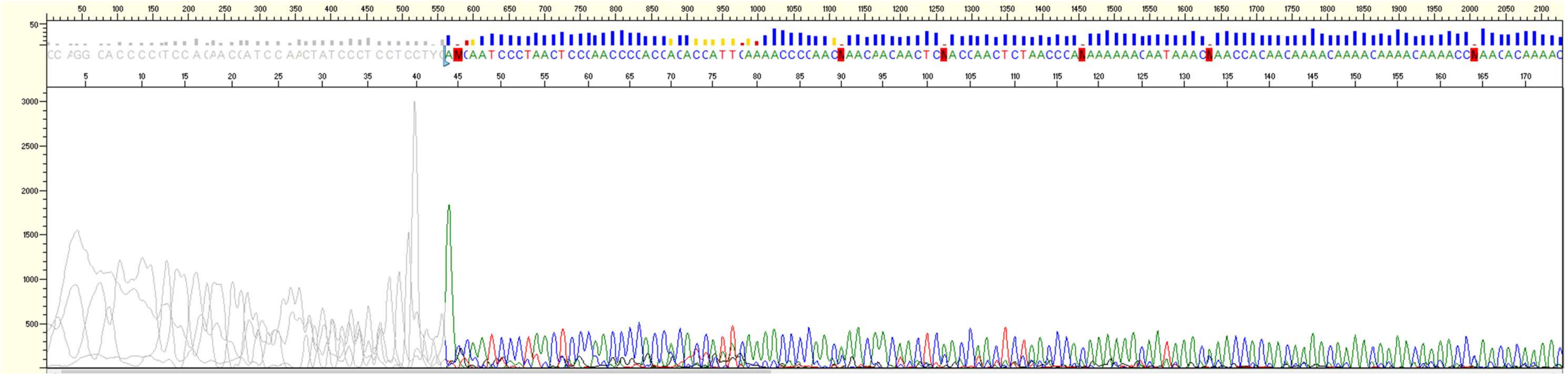

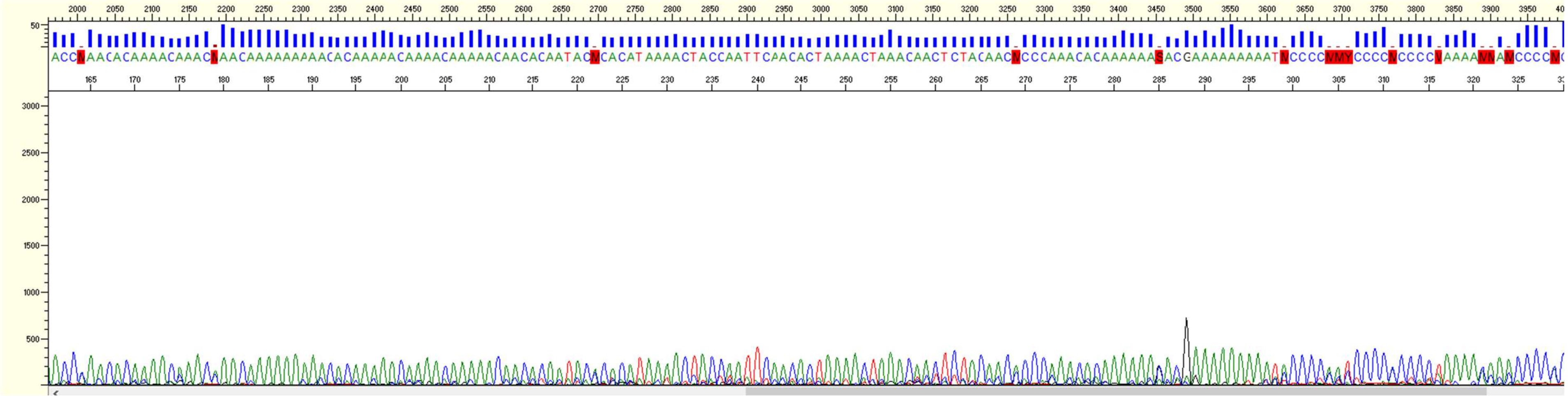

DAT

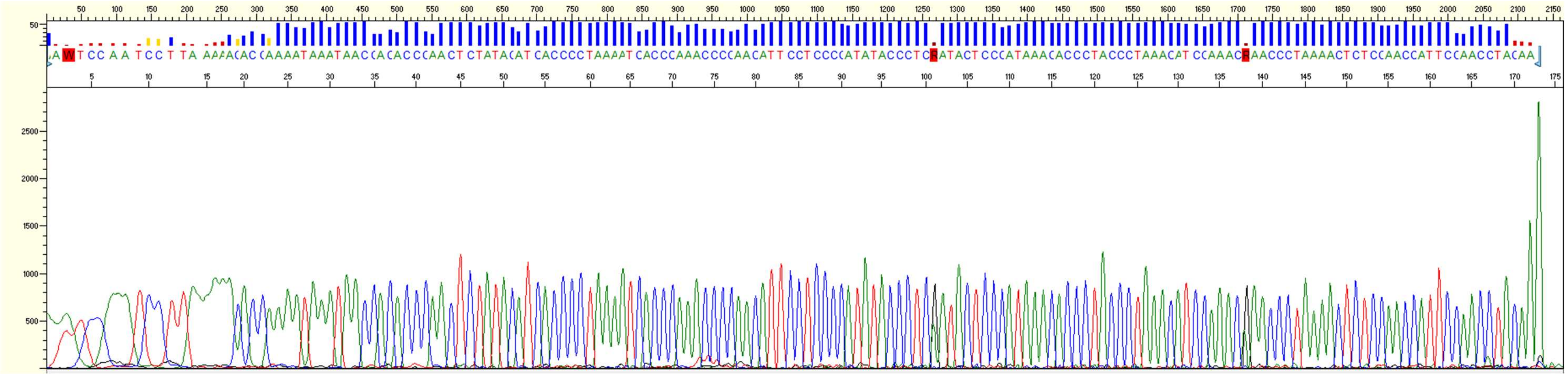

PSD 95

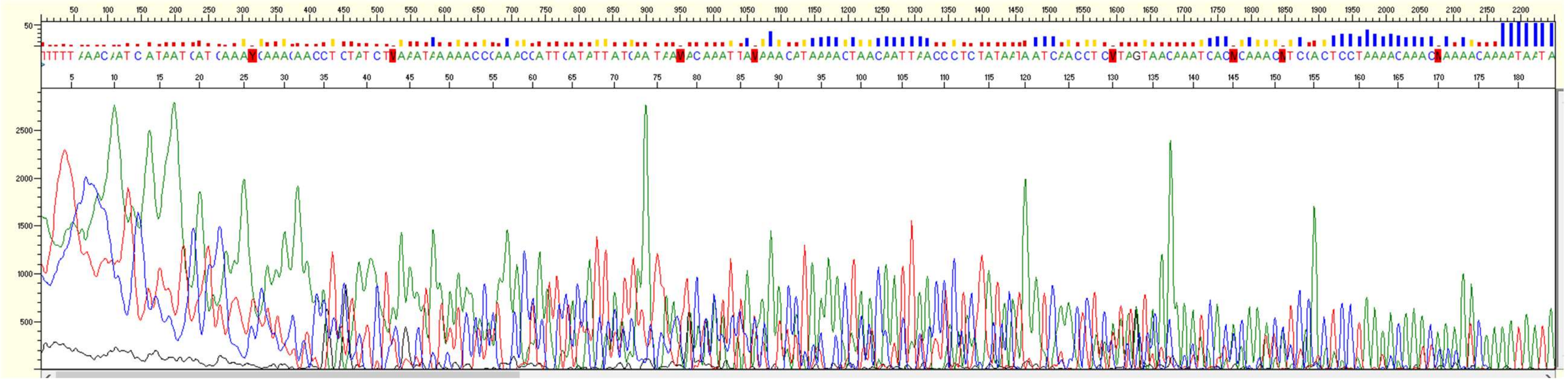

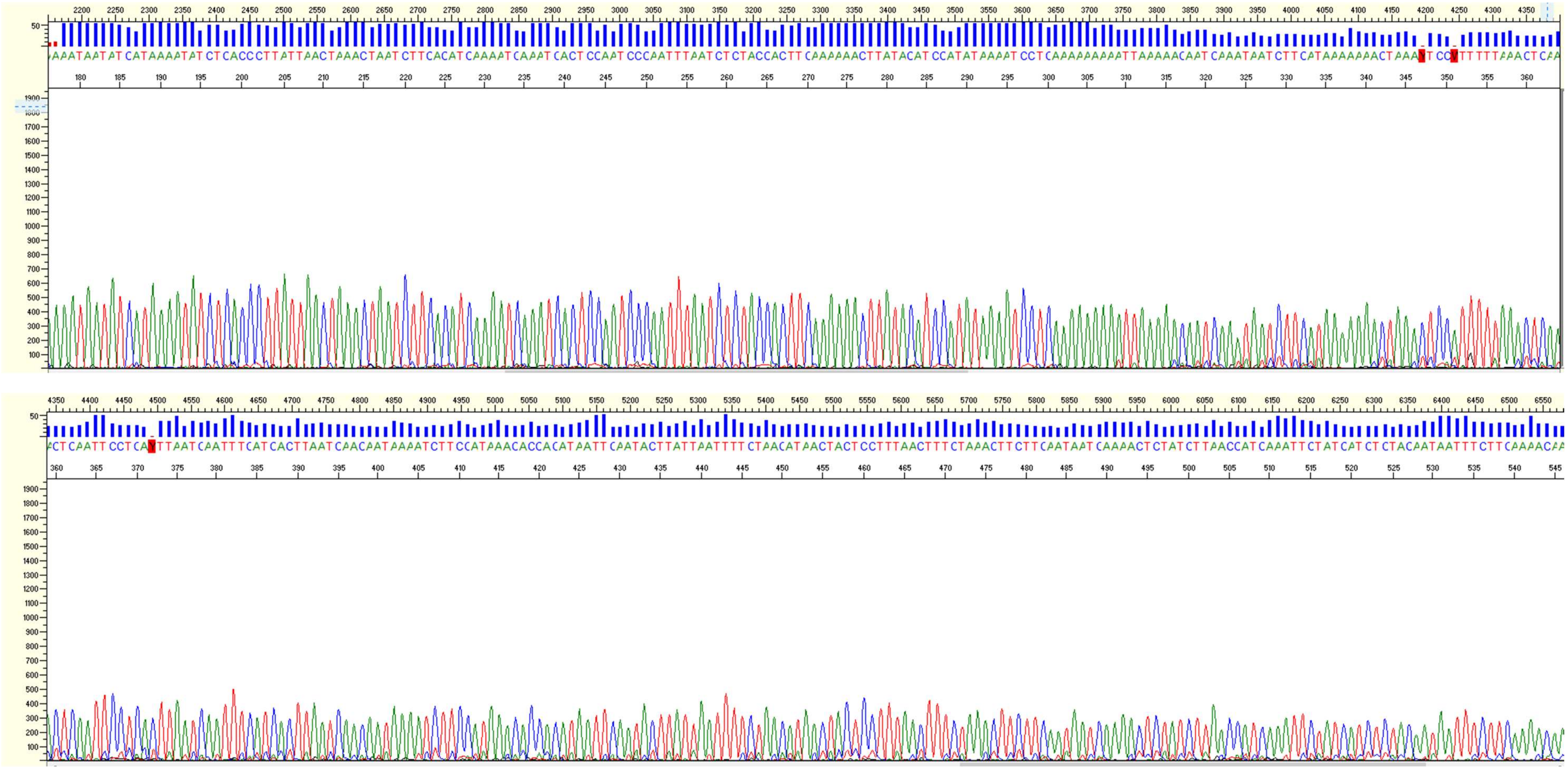

…

Suppl. Fig. 1: Representative sequencing electrograms, depicted by the Sequence Scanner Software. Initial lower sequence quality (at the end where the sequencing primer was positioned) is normal with Sanger sequencing and has been already considered during primer design in order to obtain a good sequence quality in the areas of interest.

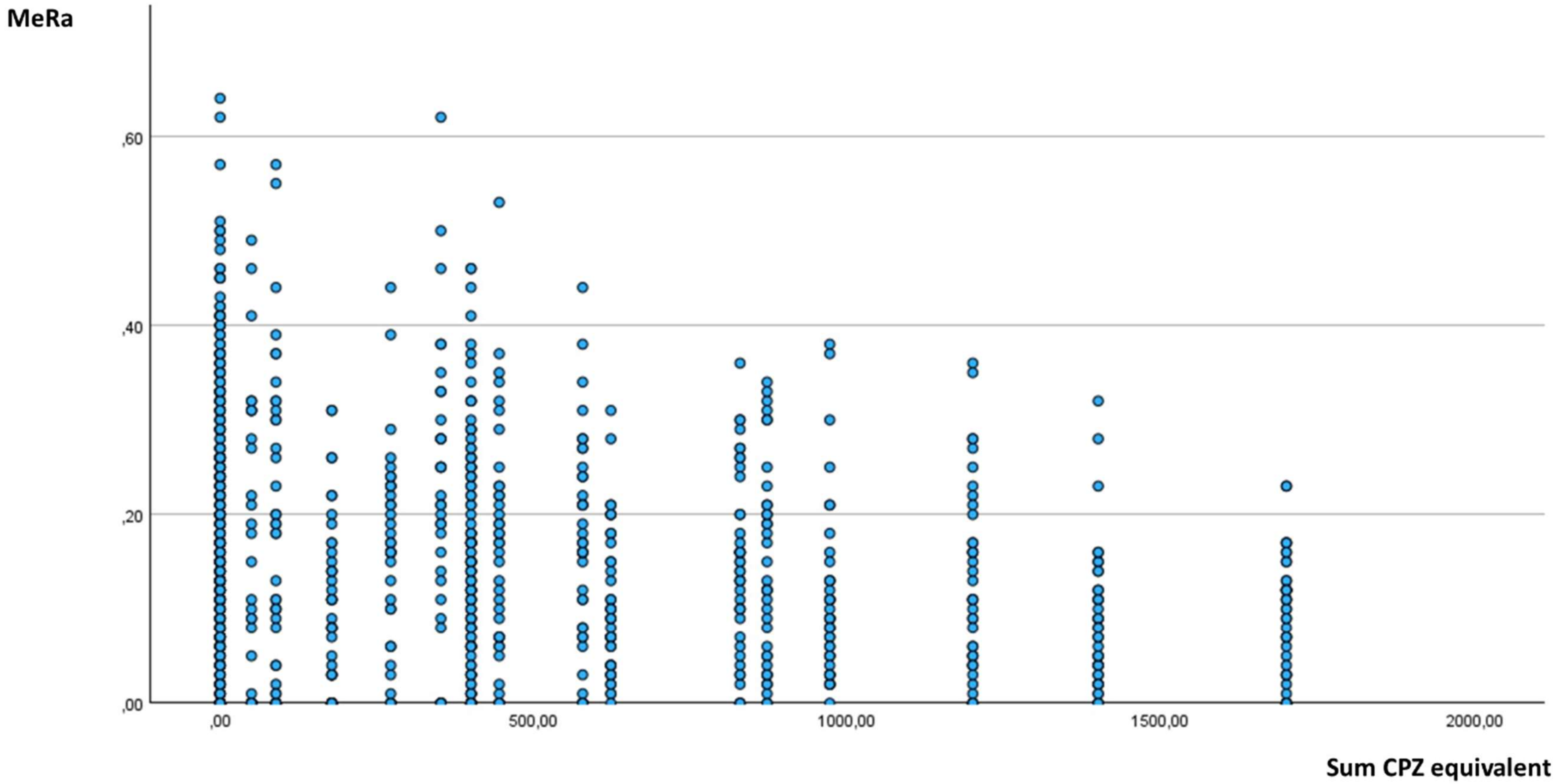

Suppl. Figure 2: Scatter plot of CPZ equivalents and DAT-Methylation Rates (MeRa, 1 corresponds to 100%) in the blood of SZ
